# Functional Divergence of Hsp90 Genetic Interactions in Biofilm and Planktonic Cellular States

**DOI:** 10.1371/journal.pone.0137947

**Published:** 2015-09-14

**Authors:** Stephanie Diezmann, Michelle D. Leach, Leah E. Cowen

**Affiliations:** Department of Molecular Genetics, University of Toronto, Toronto, Ontario, Canada; Institute of Biology Valrose, FRANCE

## Abstract

*Candida albicans* is among the most prevalent opportunistic fungal pathogens. Its capacity to cause life-threatening bloodstream infections is associated with the ability to form biofilms, which are intrinsically drug resistant reservoirs for dispersal. A key regulator of biofilm drug resistance and dispersal is the molecular chaperone Hsp90, which stabilizes many signal transducers. We previously identified 226 *C*. *albicans* Hsp90 genetic interactors under planktonic conditions, of which 56 are involved in transcriptional regulation. Six of these transcriptional regulators have previously been implicated in biofilm formation, suggesting that Hsp90 genetic interactions identified in planktonic conditions may have functional significance in biofilms. Here, we explored the relationship between Hsp90 and five of these transcription factor genetic interactors: *BCR1*, *MIG1*, *TEC1*, *TUP1*, and *UPC2*. We deleted each transcription factor gene in an Hsp90 conditional expression strain, and assessed biofilm formation and morphogenesis. Strikingly, depletion of Hsp90 conferred no additional biofilm defect in the mutants. An interaction was observed in which deletion of *BCR1* enhanced filamentation upon reduction of Hsp90 levels. Further, although Hsp90 modulates expression of *TEC1*, *TUP1*, and *UPC2* in planktonic conditions, it has no impact in biofilms. Lastly, we probed for physical interactions between Hsp90 and Tup1, whose WD40 domain suggests that it might interact with Hsp90 directly. Hsp90 and Tup1 formed a stable complex, independent of temperature or developmental state. Our results illuminate a physical interaction between Hsp90 and a key transcriptional regulator of filamentation and biofilm formation, and suggest that Hsp90 has distinct genetic interactions in planktonic and biofilm cellular states.

## Introduction

Each year, fungi kill as many individuals as malaria or tuberculosis world wide [1]. Fungal pathogenicity is determined by factors that promote survival in and on the host and cause damage [2]. One such key virulence factor is the formation of matrix-associated heterogeneous microbial communities, or biofilms [3], which are observed with the three most common invasive opportunistic fungal pathogens, including *Candida albicans* [1,4]. This member of the human microbiome [5] forms robust biofilms on medical implant devices where they are associated with mortality rates of up to 50% [6]. Hence, the clinical challenges associated with *C*. *albicans* biofilms are severe. Mature biofilms act as reservoirs for dissemination of yeast cells throughout the bloodstream [3], and the capacity to transition between yeast and filamentous forms is important for biofilm maturation [3,7] and virulence [8]. Consequently, biofilms contribute to *C*. *albicans* being the fourth most common cause of nosocomial bloodstream infections in the United States, accounting for 8–10% of bloodstream infections [9] and contributing to a 207% increase in fungal sepsis [10]. Eradicating established *C*. *albicans* biofilms is almost impossible with currently available antifungal therapies due to intrinsic drug resistance. Minimum inhibitory concentrations of mature biofilms treated with the most widely used antifungal drugs fluconazole and amphotericin B are up to 2,000 times higher than those of planktonic cells [11]. Thus, *C*. *albicans* biofilms pose a significant threat to human health necessitating investigations into their molecular and genetic control.

The essential molecular chaperone Hsp90 has emerged as a key regulator of *C*. *albicans* drug resistance and morphogenesis in both biofilm and planktonic cellular states [12–14]. Hsp90 interacts with up to 10% of the eukaryotic proteome, comprising mainly signal transducers such as kinases and transcription factors [15]. While Hsp90 is not required for *de novo* protein folding, it chaperones metastable client proteins and keeps them poised for activation [16]. The first Hsp90 client described in *C*. *albicans* is the stress-responsive protein phosphatase calcineurin [17], which enables antifungal drug resistance when stabilized by Hsp90 [12]. Since then, the kinases Cdc28 [18], Mkc1 [19], Cek1 [20], and Hog1 [21] have been shown to require Hsp90 for stability. To date, however, only one *C*. *albicans* transcription factor, the heat shock transcription factor 1 (Hsf1), has been shown to physically interact with Hsp90 [20].

To gain a more comprehensive view of the circuitry through which Hsp90 regulates key virulence traits, we previously pioneered a chemical genomic approach to map Hsp90 genetic interactions in *C*. *albicans* [21], a pathogen for which the lack of a complete sexual cycle precludes classical genetic approaches. Our screen of a transposon insertion mutant library covering ~10% of the genome revealed 226 Hsp90 chemical genetic interactors, as indicated by hypersensitivity of a mutant to Hsp90 inhibition under at least one of six planktonic conditions screened, including high temperature, mild osmotic stress, and antifungal drugs [21]. Only a few interactors were high connectivity and identified in many conditions, suggesting that they operate upstream of Hsp90 with pleiotropic effects. The majority of the interactors were low connectivity in the network and only identified in one environmental condition, suggesting that they operate downstream of Hsp90. Strikingly, 56 of the Hsp90 genetic interactors identified have functions in transcriptional regulation. Six of these transcriptional regulators have been previously implicated in biofilm formation: one high connectivity Hsp90 interactor, *FCR3*; and five low connectivity interactors, *BCR1*, *MIG1*, *TEC1*, *TUP1*, and *UPC2*. This suggests that the Hsp90 genetic interactions identified in planktonic conditions may have functional significance in the biofilm cellular state. Low connectivity interactors are enriched for downstream effectors of Hsp90 [21], motivating further analysis of the five low connectivity transcriptional regulators implicated in biofilm formation.

Here, we explored functional relationships between Hsp90 and five transcription factor genetic interactors (*BCR1*, *MIG1*, *TEC1*, *TUP1*, and *UPC2*) in biofilms and in morphogenesis, a trait important for biofilm maturation and virulence. To do so, we engineered transcription factor gene deletion mutants and assessed their capacity to form biofilms and to filament when *HSP90* expression was genetically reduced. Depletion of Hsp90 did not exacerbate the biofilm defect of the mutants. However, an interaction was observed such that deletion of *BCR1* enhanced filamentation upon reduction of Hsp90 levels. Further, we found that although depletion of Hsp90 leads to reduced expression of *TEC1*, *TUP1*, and *UPC2* in planktonic conditions, it has no impact in biofilms. Lastly, to explore the possibility of a physical link between Hsp90 and a transcriptional regulator of biofilm formation, we focused on Tup1 given that it harbors a WD40 domain, which suggests that it might interact with Hsp90 directly [22]. We identified a stable interaction between Hsp90 and Tup1 that was independent of temperature or developmental state. Taken together, our results establish a physical interaction between Hsp90 and a key regulator of morphogenesis and biofilm maturation, and suggest functional divergence in the Hsp90 genetic interaction network between planktonic and biofilm cellular states.

## Materials and Methods

### Strain maintenance

All strains used here (S1 Table) were made in the wild-type SN95 background [23] or its derivative CaLC432 (*MAL2p-HSP90/hsp90∆*) [13]. For long-term storage, yeast strains were frozen at -80°C in 25% glycerol. For routine maintenance or in preparation for experiments, strains with wild-type Hsp90 levels were grown in YPD (1% yeast extract, 2% peptone, 2% dextrose) and strains carrying the maltose-inducible promoter upstream of the sole remaining *HSP90* allele (*MAL2p-HSP90/hsp90∆*) were grown in YPM (1% yeast extract, 2% peptone, 2% maltose). To assess the effect of *HSP90* depletion, all strains were grown in YPDM (1% yeast extract, 2% peptone, 1% dextrose, 1% maltose).

### Strain construction

A PCR-based approach was used to TAP-tag or delete transcription factor genes. Both alleles were deleted by replacement with the recyclable nourseothricin (NAT) selectable marker (pJK863, a gift from J. Koehler). *Xma*I linearized plasmid at a final concentration of 0.1 ng/μl was used for PCR amplification. *TUP1* was tagged with the tandem affinity purification (TAP) tag [24]. Here, pFA-TAP-*ARG4* plasmid DNA was diluted 1:100 prior to PCR amplification [25]. Both cassettes were PCR amplified with Q5 High-Fidelity DNA Polymerase (NEB) using primers with 70 bp homology to the target locus (S2 Table). Between 100 and 400 μl ethanol precipitated PCR product were transformed into *C*. *albicans* at an optical density between OD_600_ 4 and 8 using the lithium acetate protocol [26]. Following transformation, cells were either incubated for 6 hours at 30°C in YPD or YPM prior to selection on 150 μg/ml NAT or plated immediately onto arginine drop-out medium. Transformation plates were incubated for 3 days at 30°C and transformants re-streaked onto selective media prior to genotyping PCR for the presence of the NAT or pFA-TAP-*ARG4* cassette and absence/presence of wild-type alleles (S2 Table). Transformants that tested positive for integration of the NAT marker were cultured in 5 ml YNB-BSA broth (0.17% YNB, 0.4% BSA (Sigma), 0.2% yeast extract, 2% maltose) for 3 days at 30°C to induce expression of the FLP recombinase and excise the NAT marker.

### Biofilm formation assays

Biofilm XTT metabolic activity was measured as an indicator of biofilm growth in the wild-type strain SN95, heterozygous and homozygous gene deletion mutants, and mutant strains with reduced Hsp90 levels. XTT activity was determined in two biological replicates with eight technical replicates per strain. On day 1, 96-well plates were filled with 100 μl 5% BSA in 1x PBS and incubated over night at 37°C. *C*. *albicans* strains were inoculated in 10 ml YPD or YPM and cultured at 30°C while shaking at 200 rpm overnight. On day 2, 96-well plates were washed once with 1x PBS and filled with 100 μl *C*. *albicans* cultures that were adjusted to an OD_600_ of 0.5 in 1x RPMI 1640 (2% maltose, 0.2% glucose). Following 90 minutes adherence at 37°C, non-adherent cells were removed by vacuum aspiration, wells were refilled with 100 μl 1x RPMI 1640 (2% maltose, 0.2% glucose), and plates incubated for 24 hours at 37°C. On day 3, mature biofilms were washed three times with 1x PBS and metabolic activity assessed with XTT. To do so, 10 ml XTT (BioShop Canada Inc.) stock solution (0.5 mg/ml in 1x PBS) was centrifuged for 5 minutes at 3,000 rpm and the supernatant mixed with 5 μl of 10 mM menadione in ethanol. 100 μl XTT/menadione mixture was added to each well and plates incubated for 15 minutes at 37°C, following which the OD_490_ was read for each well and the metabolic activity calculated relative to a no-cells control. Statistical significance was evaluated using GraphPad Prism 4.0.

Dry weights of biofilms grown on silicone elastomers (SEs) were determined in two biological replicates, each consisting of five technical replicates. On day 1, 1.5 cm x 2 cm SE pieces were weighed on an analytical scale and one SE was added to each well of a 6-well cell culture plate. Each SE was primed by addition of 4 ml of 5% BSA in 1x PBS and incubated overnight at 37°C without shaking. *C*. *albicans* cells were prepared by setting up a standard overnight culture in 10 ml of YPD or YPM while shaking at 200 rpm at 30°C. On Day 2, 6-well dishes were emptied by vacuum aspiration and each SE washed once with 1x PBS. Overnight *C*. *albicans* cultures were adjusted to a target OD_600_ of 0.5 in 1x RPMI 1640 (2% maltose, 0.2% glucose). 4 ml of the adjusted culture was added to five wells while the sixth well served as medium only control. Following adherence at 37°C for 90 minutes, non-adherent cells were removed by vacuum aspiration and wells were refilled with 1x RPMI 1640 (2% maltose, 0.2% glucose). On Day 3, wells were emptied carefully using a serological pipette and SEs placed onto weighted microscope slides to be air-dried for 24 hours. On Day 4, slides with SEs were weighed and dry weight increase assessed as the % weight increase between slide with SE only and slide with SE and dry biofilm.

### RNA extraction and quantitative RT-PCR

Expression levels of *HSP90*, *BCR1*, *MIG1*, *TEC1*, *UPC2* and *TUP1* were determined in biofilms and planktonic cells cultured in RPMI + 2% maltose and 0.2% glucose. To prepare sufficient cellular material for RNA extraction, biofilms were cultured in 6-well plates as described above and cells harvested from 5 wells after 48 hours of incubation at 37°C. Biofilms were allowed to mature for 48 hours, rather than 24 hours as in the XTT assay, to allow them to fill the much larger 6-well dish. Planktonic cells were started at OD_600_ 0.2 in 50 ml, cultured at 37°C while shaking at 200 rpm and harvested at an OD_600_ of ~0.8. To measure expression of *PCL1*, *PHO85* and *HMS1*, the wild-type SN95 and its derivative CaLC2676 (*bcr1∆/∆*), stationary cultures were adjusted to OD_600_ 0.2 in YPDM and incubated for 6 hours at 30°C consistent with the microscopy conditions (see below).

All cultures were harvested by centrifugation, washed once in 1x PBS, and flash frozen prior to storing pellets at -80°C. RNA was then extracted using the RNeasy mini kit (Qiagen) according to the manufacturers instructions and RNA amounts quantified using the NanoDrop 2000c. Prior to cDNA synthesis, heat-denatured RNA was separated on a 1% agarose gel and 1:5 and 1:200 RNA dilutions tested for genomic DNA contamination by PCR. 1 μg of RNA was subsequently transcribed into cDNA using the AffinityScript Multi-Temp reverse transcriptase kit (Agilent). RT-PCR was conducted on 1:300 dilutions of cDNA using the Fast SYBR Green Master Mix (Life Technologies) according to the manufacturer’s instructions on an Applied Biosystems StepOnePlus Real time PCR System qRT-PCR machine with primers designed using the Primer3web web suite (S2 Table) [27,28]. Expression levels were calculated as mean quantities of three technical replicates relative to the *GPD1* control. All qRT-PCR experiments were carried out in at least three biological replicates and statistical significance was evaluated using GraphPad Prism 4.0.

### Protein preparation and Western blot analyses

To determine Tup1 protein levels, TAP-tagged strains in the SN95 and *MAL2p-HSP90/hsp90∆* backgrounds were grown as described above for RNA extraction and qRT-PCR. Whole cell protein was extracted as described previously [20,29]. Following protein quantification using Bradford reagent (Sigma) [30], between 2 and 20 μg of whole cell protein extract was separated on 6 or 8% SDS-PAGE gels. Separated proteins were transferred to a PVDF membrane for 1 hour at 100 V at 4°C. Membranes were blocked in 5% milk in PBS containing 0.1% Tween-20 at room temperature for one hour and subsequently incubated in primary antibody. All primary antibodies were left on the membrane for one hour at room temperature. Membranes were washed with PBS-T and probed for one hour with secondary antibody dissolved in PBS-T and 5% milk. Membranes were washed in PBS-T and signals detected using an ECL western blotting kit as per the manufacturer’s instructions (Pierce).

TAP-tagged Tup1 was detected using a 1:5,000 dilution of anti-TAP tag rabbit polyclonal antibody (Thermo Scientific; CAB1001) in PBS-T plus 5% milk. To detect Hsp90, a 1:10,000 dilution of anti-Hsp90 antibody was used (courtesy of Bryan Larson) [31] in PBS-T plus 5% milk.

### Co-immunoprecipitations

These were performed as described previously [20]. Briefly, *C*. *albicans* cultures were grown to OD_600_ = 0.5, cells harvested, washed with sterile distilled H_2_O and resuspended in 1 ml lysis buffer (20 mM Tris pH 7.5, 100 mM KCl, 5 mM MgCl_2_, 20% glycerol, 1 mM PMSF (EMD Chemicals), 20 mM Na_2_MoO_4_ (Sigma), and complete EDTA-free protease inhibitor cocktail tablet (Roche Diagnostics). Cells were then disrupted by bead beating twice for 4 minutes with 7 minute breaks on ice between cycles. Lysates were centrifuged at 13,006 g for two-times 5-minutes, recovering the supernatants at each stage. The combined lysate was then cleared by centrifugation at 21,006 g for 10 minutes at 4°C and protein concentrations determined using the Bradford assay. Anti-TAP immunoprecipitations were performed by adjusting protein concentration to 1.5 mg/ml in lysis buffer containing 0.2% Tween, and incubating with Rabbit IgG agarose (Sigma #A2909) at 4°C overnight as per the manufacturer’s specifications. Unbound material was discarded, the beads were washed five times with 1 ml lysis buffer containing 0.1% Tween, and proteins were eluted by boiling in one volume of 2.5% sample buffer (125 mM Tris-HCl, pH 6.8, 5% glycerol, 2.5% SDS, 2.5% beta-mercaptoethanol, distilled H_2_O, bromophenol blue). Proteins were separated on 8% SDS-PAGE gels and probed as above.

### Morphogenesis and analysis of cell morphology by microscopy

We assessed cellular morphology of our transcription factor gene deletion mutants in the wild type (SN95) and the Hsp90 conditional expression background (*MAL2p-HSP90/hsp90∆*) (S1 Table). Overnight cultures at 30°C and 200 rpm were set up so that strains in the wild-type background were cultured in YPD and strains in the *MAL2p-HSP90/hsp90∆* background in YPM at 30°C. To observe morphology in response to Hsp90 depletion in non-filament inducing conditions, overnight cultures were then adjusted to an OD_600_ of 0.2 in YPDM medium and incubated for 6 hours at 30°C. To determine if changes in cell morphology were specific to Hsp90 depletion, transcription factor deletion mutants in the SN95 wild-type background were tested in canonical filament-inducing conditions, including 10% newborn calf serum (Invitrogen) in YPD, Lee’s Medium [32] and Spider Medium [33]. Strains grown in serum were imaged after 2 hours and those in Lee’s and Spider media after 6 hours. Images were captured using the Differential Interference Contrast setting on the Zeiss Axio Imager.MI microscope together with Axiovision software (Carl Zeiss, Inc.).

## Results

### Hsp90 interacts with key regulators of biofilm formation

**Fig 1:**
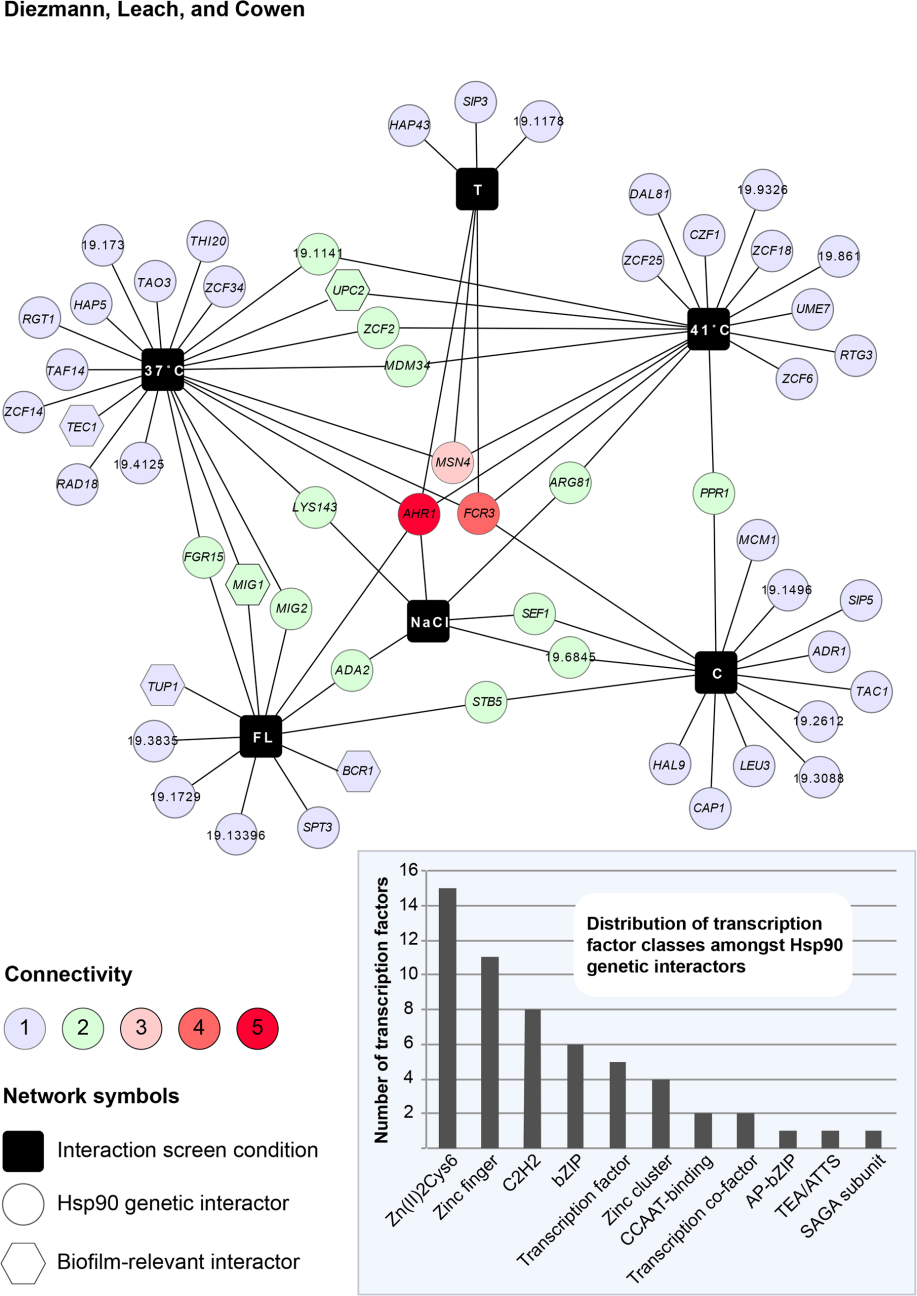
Hsp90 –transcription factors genetic interaction network. Approximately one quarter of *C*. *albicans* Hsp90 genetic interactors are transcription factors. Of the 226 genetic interactors that were previously identified in a screen of 10% of the genome [21], 56 had GO category annotations for “transcription factor”. These were further analyzed with Cytoscape 2.8.1. and transformed into the network shown here. Interactors were mapped in six environmental conditions (black squares) representing normal growth (37°C), general stresses (41°C, NaCl), and specific stresses exerted by tunicamycin (T), caspofungin (C), and fluconazole (FL). Each interactor is connected by line(s) to the condition(s) it was originally identified in. An interaction that was scored multiple times has multiple connecting lines and is located in the center of the network. The degree of connectivity is also color-coded. The five transcription factors that were further here analyzed are represented as hexagons. Hsp90 interacts with different classes of transcription factors, with representatives of the Zn(II)2Cys6 and zinc finger families being the most common interactors (inset).

To assess the extent to which Hsp90 interacts with transcriptional regulators, we searched our Hsp90 genetic interaction network for the GO category assignment ‘transcription factor’. Out of a total of 226 genetic interactors [21], 56 fit this GO category and were further analyzed. Network analysis depicts these Hsp90 interactors with respect to the experimental conditions in which each of them was originally identified (Fig 1). The resulting Hsp90 –transcription factor genetic interaction network exhibits similar properties to our full network, with only a few interactors identified in the majority of conditions. The two high-connectivity interactors that were identified in the majority of conditions, *AHR1* and *FCR3*, were previously shown to regulate *HSP90* expression [21] or to be required for fluconazole resistance [34] and biofilm adherence [35]. The vast majority of interactors were only identified in one or two environmental conditions, suggesting that they operate downstream of Hsp90.

Based on computational and experimental analyses, this set of 56 genes represents a diverse array of transcription factor classes, many of which have zinc finger domains (Fig 1 inset) (www.candidagenome.org access: May 29^th^, 2015). While 46% of the Hsp90-interacting transcription factor genes are still considered ‘putative’ or ‘uncharacterized’, others have well-defined functions in biologically diverse processes such as cell wall integrity (*ADA2*), low-iron response (*HAP43*), drug response (*TAC1*), oxidative stress response (*CAP1*), or transcriptional regulation, as with the SAGA complex (*SPT3*). Five of the low-connectivity transcription factors that Hsp90 interacts with play some role in biofilm development: *BCR1*, *MIG1*, *TEC1*, *TUP1*, and *UPC2* (Fig 1). These five were identified in the 37°C, 41°C, and fluconazole screens. Growth under basal conditions of 37°C, the prerequisite temperature for biofilm formation, led to the identification of *TEC1*, *MIG1*, and *UPC2*. Growth at an elevated temperature of 41°C, which permits biofilm formation but renders biofilms more drug susceptible [36], yielded *UPC2*. While, exposure to 0.1 μg/ml fluconazole, to which biofilms are resistant [11], resulted in identification of *MIG1*, *TUP1*, and *BCR1*. These transcription factors have established roles in different aspects of biofilm formation: Bcr1, which is regulated by Tec1, is essential for biofilm initiation and adherence [37]; the transcriptional co-repressor of filamentation Tup1 [38] is down-regulated in mature biofilms [39]; the catabolic repressor Mig1 is overexpressed in biofilms [40]; and the transcriptional regulator of ergosterol biosynthesis genes Upc2 requires Tye7 for normal expression in biofilms [41]. Given their status as low-connectivity interactors in the Hsp90 genetic interaction network, we hypothesized that transcriptional regulators may illuminate circuitry through which Hsp90 influences biofilm biology.

### Reduction of Hsp90 levels in transcription factor mutants does not exacerbate biofilm formation defects

**Fig 2:**
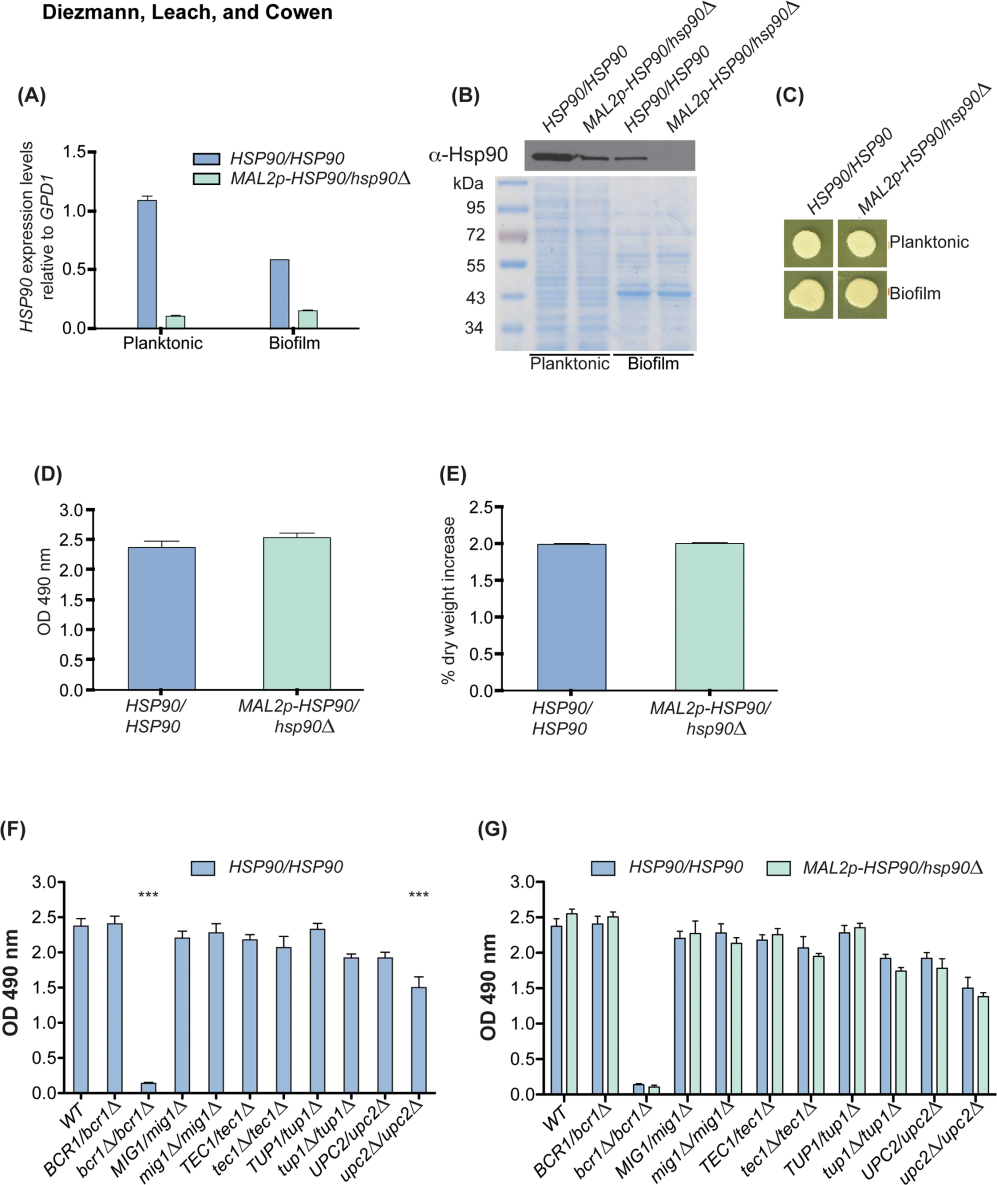
*HSP90* genetic interactions identified in planktonic conditions do not impact biofilm formation. To genetically deplete *HSP90* the maltose-inducible promoter replaced the native promoter of the sole remaining *HSP90* allele. The wild type and *MAL2p-HSP90/hsp90* strain were then grown in RPMI 1640 (2% maltose, 0.2% glucose) planktonically and as biofilms prior to quantifying mRNA and protein levels. **(A)** *HSP90* gene expression levels differ in the wild-type strain by ~50% between planktonic and biofilm cultures. In the Hsp90 depleted strain, *HSP90* is reduced in both conditions. **(B)** Western blot analysis of Hsp90 levels confirmed changes in Hsp90 protein levels. A Coomassie Blue stained 10% SDS-PAGE gel loaded with 5 μg of whole cell protein extract served as loading control. **(C)** Genetic depletion of *HSP90* using the maltose inducible promoter does not affect cell viability in strains grown planktonically or as biofilms. Biofilms were cultivated at 37°C in RPMI (2% maltose, 0.2% glucose). To ensure cells used for protein and RNA extraction were still alive following prolonged *HSP90* repression, cultures were spotted onto YPM, which promotes growth of strains with the *MAL2* promoter. Neither biofilm metabolic activity **(D)** nor dry weight **(E)** are affected by genetic depletion of Hsp90. **(F)** Homozygous deletion of *BCR1* and *UPC2* results in significantly reduced XTT conversion rates (*** = P < 0.001). **(G)** Further depleting *HSP90* in the transcription factor deletion mutants has no effect on metabolic activity as measured by XTT conversion rates.

Before embarking on functional characterization of the Hsp90-transcription factor relationships and their impact on biofilm formation, we sought to verify our *HSP90* depletion system and quantify biofilm growth in response to *HSP90* depletion. To reduce *HSP90* expression levels, we utilized a conditional expression system in which one allele of *HSP90* is deleted and the promoter of the remaining allele is replaced with the *MAL2* promoter (*MAL2p*), which induces transcription in the presence of maltose and represses transcription in the presence of glucose [13]. For long-term repression of *HSP90* without detrimental effects on the cell, strains were grown in 1x RPMI 1640 containing 2% maltose and 0.2% glucose. This led to an ~80% decrease in *HSP90* levels in planktonic cells (Fig 2A). Strikingly, when we measured *HSP90* levels in biofilms, *HSP90* expression from the native promoter was ~50% lower compared to that observed in planktonic cells. Expression of *HSP90* from the *MAL2* promoter was comparable between planktonic and biofilm cellular states. We further corroborated our findings by analysis of Hsp90 protein levels. Hsp90 antibody staining of whole cell protein extracts revealed reduced Hsp90 levels similar to those observed for *HSP90* transcript levels (Fig 2B). To ensure cell viability despite severe reduction of Hsp90 levels, planktonic and biofilm cell suspensions were spotted onto YPM and incubated for 48 h. Spot assays indicated vigorous growth in cells with wild-type and genetically reduced Hsp90 levels independent of the developmental program (Fig 2C). Next, we aimed to determine whether reducing Hsp90 levels using the *MAL2* promoter would affect biofilm metabolic activity or dry weight in cells cultured in RPMI 1640 (2% maltose, 0.2% glucose) plus maltose. Reduced Hsp90 levels did not affect biofilm growth (Fig 2D and 2E), as expected based on our previous analysis with a different conditional expression system [14]. This provides an approach by which to assess if Hsp90 genetic interactions identified in planktonic cells have functional relevance in the biofilm cellular state.

Upon establishing a reliable Hsp90 depletion system, we sought to determine if reducing *HSP90* in transcription factor mutants significantly affected their biofilm development. We first tested heterozygous and homozygous transcription factor gene deletion mutants for their biofilm metabolic activity (Fig 2F). Only the homozygous deletion mutants of *BCR1* and *UPC2* were unable to form fully active biofilms (Fig 2F). This result confirms *BCR1*’s involvement in biofilm formation [37], while establishing a new role for *UPC2* in *C*. *albicans* biofilm development. *UPC2* may not have been identified in previous screens for regulators of biofilm formation based on the different culture conditions used. Differences in culture conditions may also account for why we did not identify an impact of *TEC1* deletion on biofilm development, despite Tec1 having multiple established roles in biofilm physiology [42–44]. Intriguingly, reducing Hsp90 levels did not further compromise biofilm formation in any of the transcription factor mutant strains tested (Fig 2G). This suggests that Hsp90 may have distinct genetic interactions in biofilm compared to planktonic cellular states, and motivated further analysis of functional relationships between Hsp90 and the transcription factors.

### Deletion of *BCR1* results in hyperfilamentation upon reduction in Hsp90 levels

**Fig 3:**
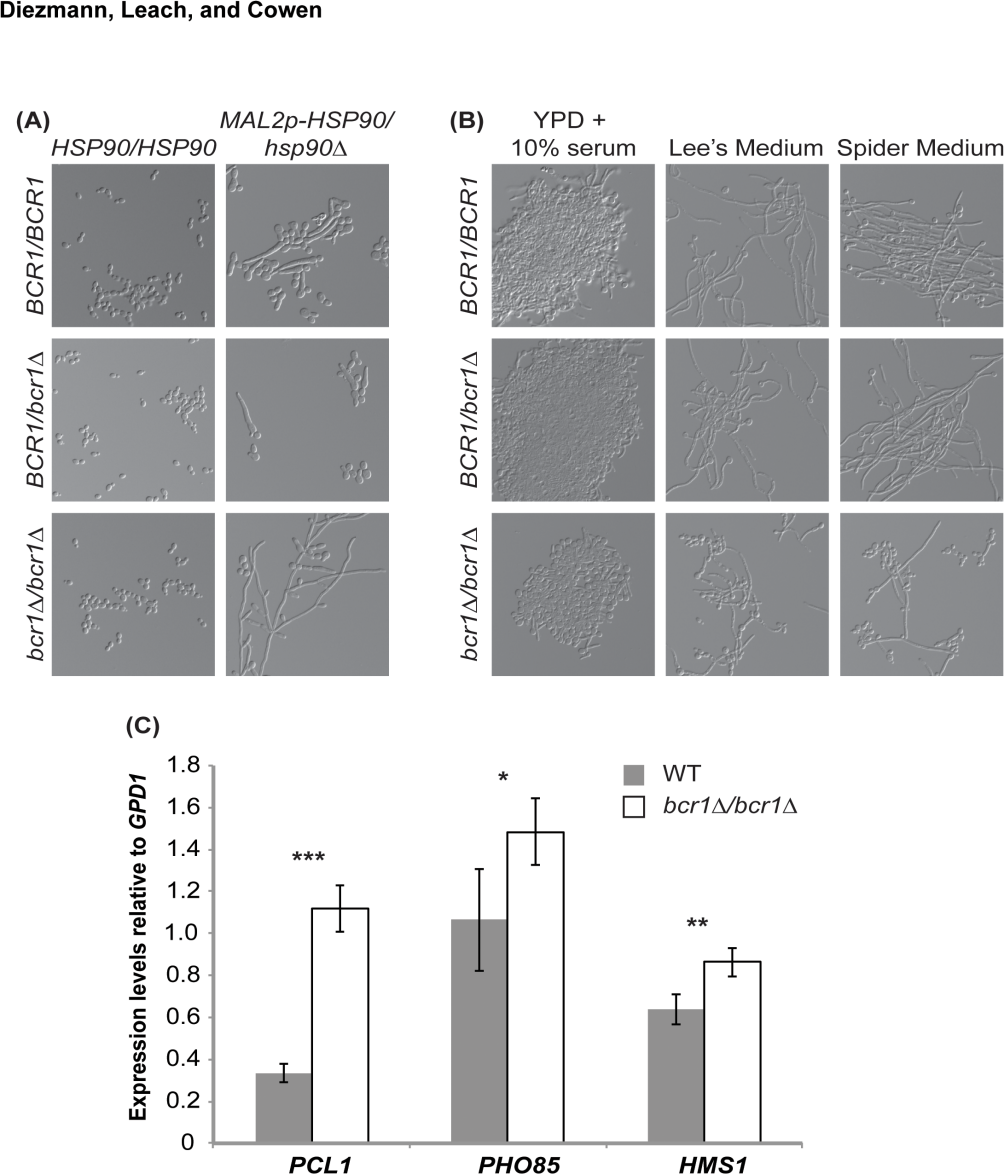
Bcr1 antagonizes Hsp90’s effect on filamentation. **(A)** Growing strains with wild type and reduced levels of Hsp90 under conditions that favour yeast growth (YPDM, 30°C) results in filamentation upon Hsp90 compromise. Deletion of *BCR1* enhances filamentation induced by Hsp90 depletion. **(B)** Deletion of *BCR1* alone does not affect filamentation under canonical filament-inducing conditions. Cells were imaged after 2 hours (YPD + 10% serum) and 6 hours (Lee’s and Spider media). **(C)** qRT-PCR measuring expression levels of *PCL1*, *PHO85*, and *HMS1* in wild type and *bcr1∆/∆* mutant strains grown in non-filament inducing conditions at 30°C. Bcr1 represses expression of genes in the *PHO85*-*PCL1*-*HMS1* pathway (* = P < 0.05, ** = P < 0.01, *** = P < 0.001).

To determine if any of the transcription factors modulate filamentation induced upon depletion of Hsp90, we cultured the *HSP90* conditional expression strains in glucose-containing medium (YPDM) at 30°C for short-term transcriptional repression of *HSP90* and monitored cellular morphology by microscopy. In parallel, we monitored morphology of the transcription factor deletion mutants with native *HSP90* levels as a control. The *tup1∆/∆* deletion mutant was the only homozygous deletion mutant that filamented in the absence of any inducing cue (S1 Fig); this phenotype was expected given the established role of Tup1 as a transcriptional repressor of filamentation [45]. Homozygous deletion of the transcription factor genes did not alter morphology of the *MAL2p-HSP90/hsp90∆* (S1 Fig). Only simultaneous homozygous deletion of *BCR1* and depletion of *HSP90* resulted in a phenotype different from either single genetic lesion (Fig 3A, S2 Fig). In the absence of Bcr1, the *MAL2p-HSP90/hsp90∆* strain exhibited elongated filaments upon Hsp90 depletion (Fig 3A). This result illuminates a new facet of the *HSP90* –*BCR1* genetic interaction, and suggests that Bcr1 antagonizes Hsp90’s repressive effects on morphogenetic programs.

Next, we tested whether Bcr1 antagonizes filamentation induced by other cues, or whether the effect is specific to Hsp90 compromise. To do so, we cultured the wild-type strain along with the *BCR1* heterozygous and homozygous deletion mutants in filament-inducing conditions including 10% serum, Lee’s Medium, and Spider Medium, and imaged cells after two or six hours at 37°C. Loss of *BCR1* did not enhance filamentation in response to these cues; rather, filaments were generally shorter and present at a reduced frequency in the population (Fig 3B). Thus, although Bcr1 antagonizes filamentation induced upon Hsp90 depletion, it enhances filamentation in opaque cells [46], and is a positive regulator of hyphal-specific genes [37]; suggesting divergent roles in regulating morphogenesis in response to different cues.

To explore the mechanism by which Bcr1 exerts a repressive effect on morphogenetic circuitry specifically required for response to Hsp90 compromise, we turned to the pathway composed of the cyclin Pcl1, cyclin-dependent kinase Pho85, and transcription factor Hms1. We previously established that signaling through the Pho85-Pcl1-Hms1 pathway is required for filamentation in response to Hsp90 inhibition but not in response to serum, Lee’s Medium, or Spider Medium [47]. To determine if Bcr1 modulates expression of components of this pathway, we measured *PHO85*, *PCL1*, and *HMS1* transcript levels in wild type and *bcr1∆/∆* mutant cells grown in YPD at 30°C. Transcript levels of all three genes were significantly higher in the *bcr1∆/∆* mutant compared to the wild type (Fig 3C). This suggests that Bcr1 blocks filamentation in response to Hsp90 depletion by repressing the Pho85-Pcl1-Hms1 pathway.

### Hsp90 activates transcription factor gene expression in planktonic cells but not in biofilms

**Fig 4:**
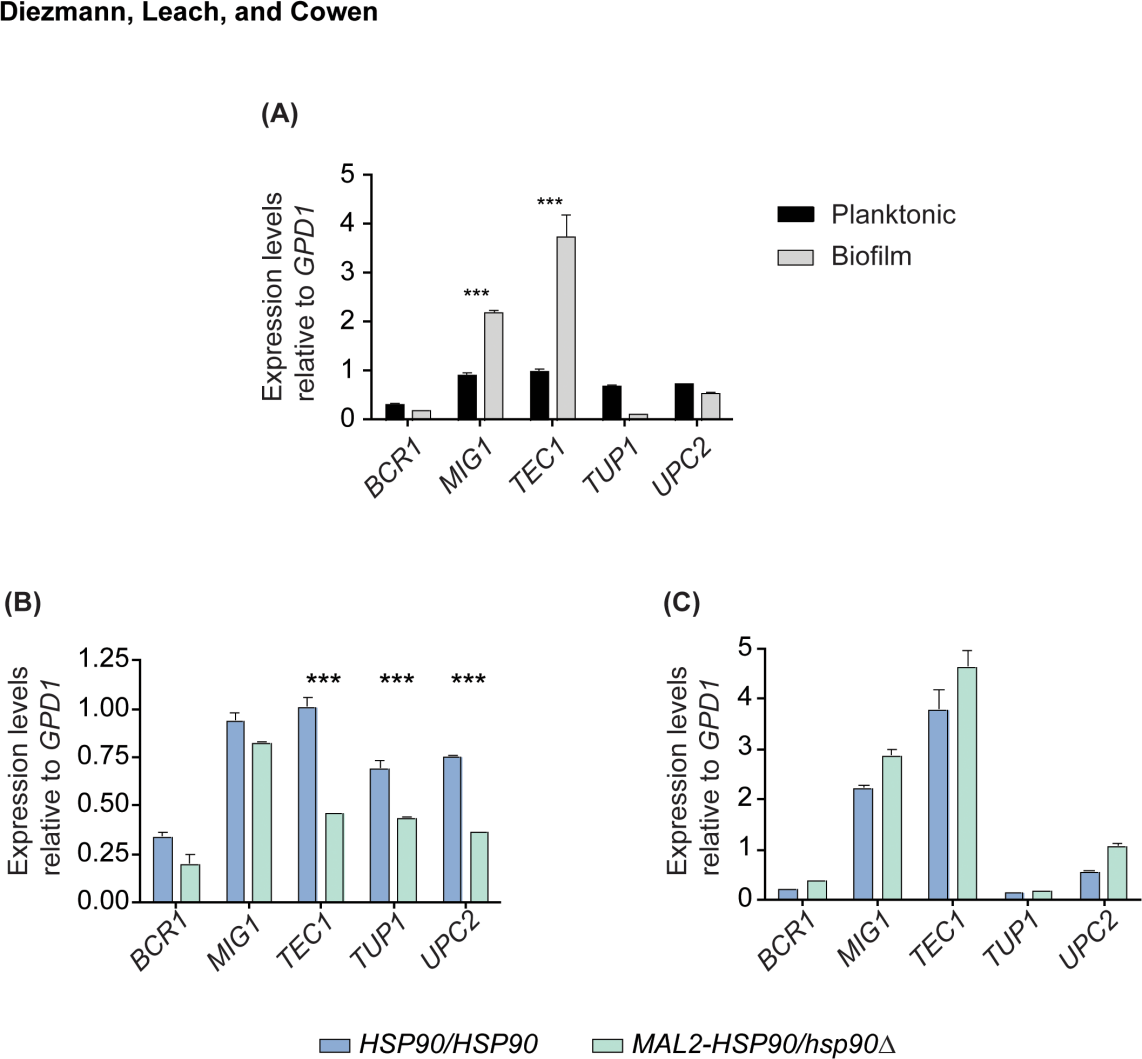
Hsp90 is a positive regulator of transcription factor gene expression in planktonic cells. **(A)** *TEC1* and *MIG1* are significantly up-regulated in biofilms compared to their planktonic counterparts. **(B)** Reduction of Hsp90 levels leads to decreased expression of *TEC1*, *TUP1*, and *UPC2* during planktonic growth at 30°C with shaking at 200 rpm (*** = P < 0.001). **(C)** Reduction of Hsp90 does not affect expression of the transcription factor genes during biofilm growth (37°C, static).

Gene expression profiles differ between planktonic cells and biofilms, with transcription factors being differentially regulated in both cellular states [48]. Given the pivotal role of transcription factor gene expression in biofilm formation, we hypothesized that expression of the five factors investigated here would change between planktonic cells and biofilms. We then aimed to determine if Hsp90 is required for changes in transcription factor gene expression. To do so, RNA from mature biofilms and planktonic cells was extracted and analyzed by qRT-PCR. Only two of our five key transcription factors displayed differential expression between the cellular states; *MIG1* [40] and *TEC1* were two- to four-fold up-regulated in biofilms relative to planktonic cells (Fig 4A). We found that depletion of Hsp90 functioned as a positive regulator of transcription factor gene expression during planktonic growth, but not in biofilms (Fig 4B and 4C). Depleting Hsp90 in planktonic cells resulted in significantly reduced *TEC1*, *TUP1*, and *UPC2* gene expression, indicating that Hsp90 is required for full expression of these genes. However, loss of *HSP90* had no effect on gene expression of our key transcription factors during biofilm growth. That this functional relationship between Hsp90 and the transcription factor genes is specific to planktonic cells reinforces the notion that genetic interactions are contingent on developmental state.

### Tup1 and Hsp90 form a stable interaction independent of environment or developmental state

**Fig 5:**
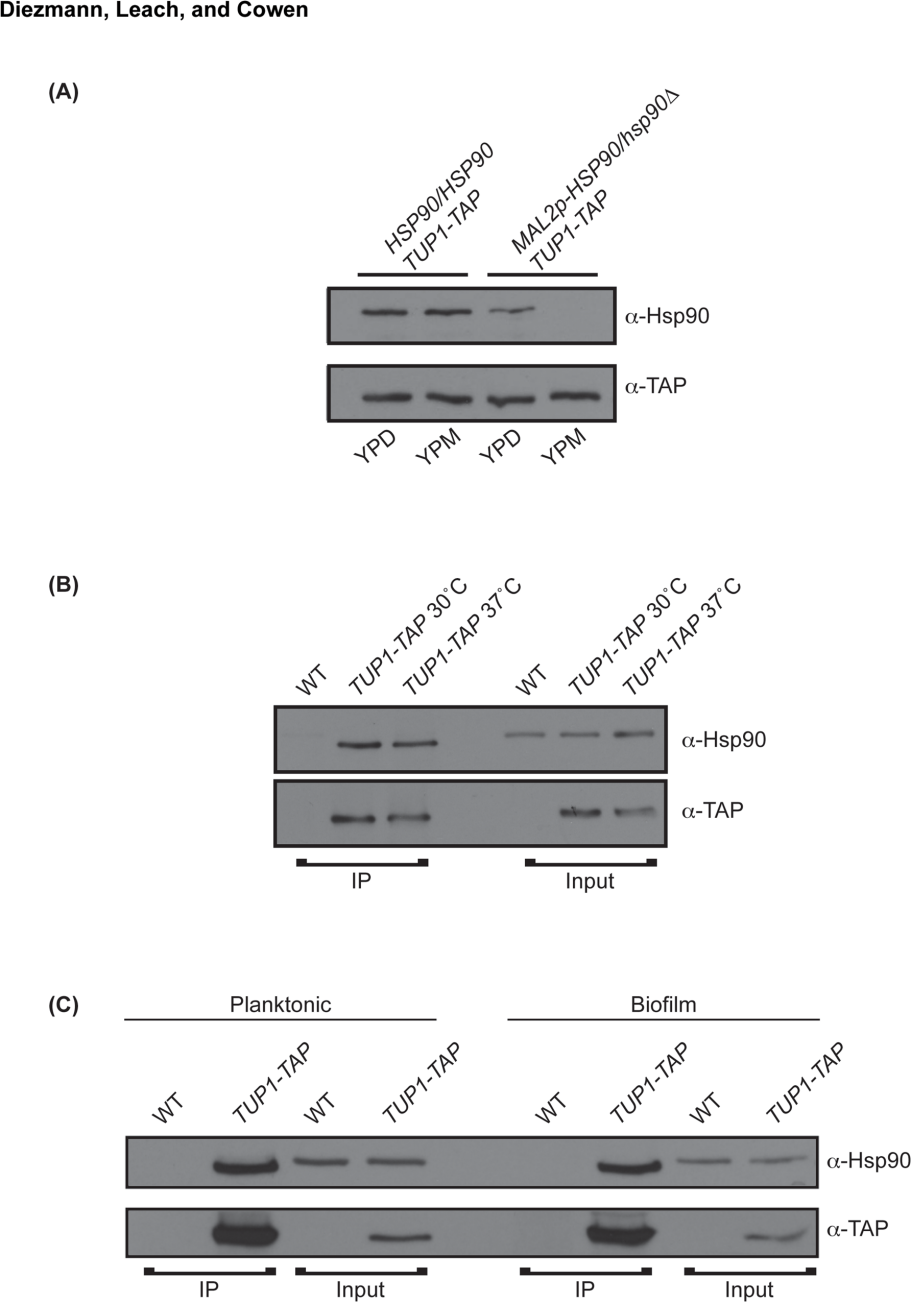
Hsp90 and Tup1 have a stable interaction that is independent of growth temperature or developmental state. **(A)** Antibody staining of whole cell protein extracts of planktonic cells grown at 30°C demonstrates that genetic depletion of *HSP90* using the *MAL2p* promoter does not affect Tup1 protein levels. **(B)** Immunoprecipitated (IP) and input samples from wild type and *TUP1-TAP* tagged strains grown at 30°C and 37°C. TAP-tagged Tup1 was pulled down using IgG agarose and blots were probed with anti-Hsp90 and anti-TAP antibodies. Enrichment of Hsp90 in the IP sample indicates that the physical interaction remains stable during standard growth (30°C) and in biofilm-inducing temperatures (37°C). **(C)** Hsp90 and Tup1 interact during biofilm and planktonic growth. Hsp90 is enriched in the immunoprecipitated *TUP1-TAP* strain relative to the input control.

The regulation of *TEC1*, *TUP1*, and *UPC2* expression in planktonic conditions confirms that Hsp90 genetically interacts with these three transcription factors, as predicted from the chemical genomic screen originally conducted in planktonic cells [21]. Although, overlap between genetic and physical interactors of Hsp90 is limited [15], Tup1’s WD40 domain has recently been shown to be an Hsp90 client protein fold [49]. Thus, we asked if Hsp90 and Tup1 physically interact, and if Hsp90 modulates stability of Tup1. To do so, we measured Tup1 protein levels in cells with wild type and reduced levels of Hsp90, and conducted a series of co-immunoprecipitation experiments to assess the presence of a physical interaction in different temperatures and developmental states. To determine if Hsp90 is required for Tup1 protein stability, one *TUP1* allele was TAP tagged in the wild type and the *MAL2p-HSP90* strain, and whole cell proteins were extracted from cells grown at 30°C in YPD. Tup1 protein levels did not decrease when Hsp90 was genetically depleted, suggesting that Hsp90 is not required for Tup1 stability (Fig 5A). Although Tup1 protein levels do not depend on Hsp90, the chaperone may be required for Tup1 maturation. We thus investigated the possibility of an Hsp90-Tup1 physical interaction by co-immunoprecipitating proteins from cells grown at standard temperature (30°C) and at physiological or biofilm-inducing temperature (37°C). At both temperatures, immunoprecipitation of TAP tagged Tup1 with IgG agarose co-purified both TAP-tagged Tup1 and wild-type Hsp90 (Fig 5B). For the control strain lacking the tagged *TUP1* allele, Hsp90 was present in the input but was not immunoprecipitated (Fig 5B). Since temperature did not appear to affect the Hsp90- Tup1 interaction, we next tested if it may be contingent on cellular state. To do so, proteins from planktonic and biofilm cells cultured at 37°C were co-immunoprecipitated and processed. Again, Hsp90 co-purified with Tup1, indicating that their interaction is independent of developmental state (Fig 5C). This suggests that while Hsp90 is not required for Tup1 stability, some portion of the Tup1 pool interacts with Hsp90. Thus, Tup1 is a novel Hsp90 interactor whose expression is regulated by Hsp90 in planktonic but not biofilm cells.

## Discussion

Our analysis of functional relationships between Hsp90 and transcription factor genetic interactors reveals a stable physical interaction between Hsp90 and a key morphogenetic regulator, and suggests that the Hsp90 genetic interaction network may be exquisitely contingent upon cellular state. Genetic interactions between *HSP90* and the biofilm-related transcription factor genes *BCR1*, *MIG1*, *TEC1*, *TUP1*, and *UPC2* that were identified in planktonic conditions (Fig 1), had little impact on fitness of biofilms, such that depletion of *HSP90* did not enhance any biofilm defect of the transcription factor deletion mutants (Fig 2). In contrast, an additional interaction was observed in planktonic conditions, such that deletion of *BCR1* enhanced filamentation induced by reduction of Hsp90 levels (Fig 3). Consistent with this divergence between interactions in different cellular states, depletion of Hsp90 had no impact on expression of the five transcription factor genes in biofilms, but led to reduced expression of *TEC1*, *TUP1*, and *UPC2* in planktonic conditions (Fig 4), suggesting that Hsp90 modulates a core transcriptional regulatory circuit during specific developmental programs.

As a central hub of protein homeostasis and regulatory circuitry, Hsp90 is poised to exert a profound impact on transcriptional regulation. There are at least three ways in which Hsp90 tunes transcription [50]. First, Hsp90 modulates activation of diverse transcription factors, thereby enabling transcriptional responses to specific cues [51]. A classic example is with the transcription factor Hsf1; Hsp90 physically interacts with Hsf1 and exerts a repressive effect on this global regulator of the heat shock response, such that compromise of Hsp90 function leads to Hsf1 activation and induction of heat shock genes [20,52,53]. Second, Hsp90 modulates epigenetic gene regulation as it controls the activity of chromatin remodeling factors and epigenetic modifiers, enabling transcriptional responses [50]. This is illustrated by the Hsp90 co-factors Tah1 and Pih1 in *S*. *cerevisiae*, which physically interact with Rvb1/Rvb2, core components of several chromatin remodeling factors [15]. Third, Hsp90 is required for removal of nucleosomes from promoter regions of specific genes, thereby enhancing transcriptional activation. In *S*. *cerevisiae*, deletion of the Hsp90 gene *HSC82* delays nucleosome removal from the *GAL1* promoter [54]. Our finding that Hsp90 activates the expression of some but not all of the transcription factor genes in planktonic conditions (Fig 4), suggests that the transcriptional control is specific rather than global in nature. Hsp90 could modulate transcription of these biofilm regulators via multiple mechanisms, with pleiotropic effects expected given that they participate in a highly interconnected network controlling biofilm development [55]. Hsp90 levels are intimately coupled to developmental programs, as they increase during biofilm initiation [56], and decrease accompanying maturation (Fig 2). Beyond expression level, Hsp90 function is also environmentally contingent, such that elevated temperature and other conditions that cause global protein misfolding can create a cellular demand for Hsp90 that exceeds its functional capacity [57]. Thus, Hsp90 provides a powerful mechanism to tune transcriptional circuits in response to environmental conditions and developmental cues.

The interaction between Hsp90 and transcription factors or other client proteins can have multiple functional consequences. Classically, client proteins are defined by a physical interaction with Hsp90 and by activity that is contingent upon Hsp90 function, such that inhibition of Hsp90 leads to reduced client protein activity and often client protein degradation [49,58]. Client proteins often interact dynamically with Hsp90 and co-chaperones, until they achieve their final maturation, localization, or activation. In *C*. *albicans*, several Hsp90 client proteins have been characterized, as with the protein phosphatase calcineurin and the terminal MAPK of the cell wall integrity pathway Mkc1 [12,19]. Hsp90 stabilizes these regulators and enables their activation in response to stress in planktonic conditions. Strikingly, depletion of Hsp90 has no impact on their stability in biofilm conditions, suggesting that compensatory mechanisms may buffer client protein stability in the biofilm cellular state [14]. The only transcription factor that has been established as an Hsp90 client protein in *C*. *albicans* is Hsf1 [20]. Unlike with many client proteins, Hsp90 interacts robustly with Hsf1 under basal and stress conditions, and Hsp90 exerts a repressive effect on Hsf1 function. Our findings that Hsp90 and Tup1 stably interact in both planktonic and biofilm conditions (Fig 5), suggest that Tup1 may be a novel Hsp90 client protein. Depletion of Hsp90 does not lead to destabilization of Tup1, thus Hsp90 may be involved in localization of Tup1 or modulation of its DNA binding activity. Given that Tup1 works in concert with Nrg1 and Mig1 to regulate expression of the *ALS* and *SAP* gene families [59], the physical interaction between Hsp90 and Tup1 links Hsp90 to key regulatory circuitry controlling *C*. *albicans* adherence [60], invasion [61], and virulence [62].

The link between Hsp90 and transcriptional circuitry controlling traits relevant for *C*. *albicans* pathogenesis further extends to the transcriptional regulator Bcr1 and the capacity for morphological transitions between yeast and filamentous growth. Bcr1 is a downstream component of the filamentation regulatory network as it is upregulated in filaments and promotes adherence through Als3 [3,37]. In addition to its role as a positive regulator coupling the expression of cell surface genes to filamentation, it has emerged as a repressor of filamentation in the *C*. *albicans* opaque cellular state [63]. Opaque cells represent a distinct epigenetic switch state from the standard white cells, with white and opaque cells exhibiting distinct programs of filamentous growth that are induced by different cues [64]. Our finding that deletion of *BCR1* enhances filamentation that is induced upon depletion of Hsp90 (Fig 3), suggests that Bcr1 may antagonize Hsp90’s repressive effects on morphogenetic programs. Hsp90 is known to control morphogenesis through core pathways involved in filamentation in response to many cues, as together with the co-chaperone Sgt1, it interacts with the adenylyl cyclase Cyr1 and represses Ras1-PKA signaling [65]. Hsp90 also regulates morphogenesis through circuitry that has specialized for responding only to specific filament-inducing cues, as with signaling through the cyclin Pcl1, cyclin-dependent kinase Pho85, and transcription factor Hms1 [47]. Consistent with deletion of *BCR1* specifically enhancing filamentation induced by Hsp90 depletion in white cells, Bcr1 exerts a repressive effect on expression of *PCL1*, *PHO85*, and *HMS1* (Fig 3). With the recent development of pooled screening approaches that enable high-throughput genome-scale analysis of *C*. *albicans* morphogenesis [66], we are now poised to elucidate the complex genetic control of filamentation programs that are induced by diverse host relevant cues.

The divergence in filamentation programs induced by different cues and cellular states resonates with the divergence in genetic interaction networks in response to changes in the external or internal environment. The vast majority of Hsp90 genetic interactions are contingent on the environment in which they are measured. This is likely a consequence of condition-specific functions of Hsp90 or the genetic interactor. Consistent with this model, divergence in Hsp90 chemical genetic interactors in *S*. *cerevisiae* in response to basal and stress conditions reflects condition-specific functions of Hsp90 [67]. Under basal conditions, Hsp90 has key roles in protein transport and the secretory pathway, as well as stabilizing oligomeric complexes, while in stress conditions, Hsp90 is required for control of cell cycle, meiosis, and cytokinesis [67]. Divergence in genetic interactions in response to environmental conditions or cellular states can also arise from expression or activation of compensatory pathways in a condition-specific manner. In support of this model, it is clear that the majority of transcription factors contain intrinsically disordered domains whose functions are modulated by environmental and developmental contexts, and further controlled by post-translational modification [68]. Our findings that the Hsp90 genetic interaction network may be strikingly different between planktonic and biofilm cellular states, motivates analysis of genetic interaction networks directly in biofilms to elucidate the circuitry governing morphogenesis and drug resistance. Our work complements the emerging paradigm that genetic interaction networks are dynamic and responsive to changes in environmental conditions and development states in diverse species.

## Supporting Information

S1 Table Strains used in this study. (DOCX)

S2 Table Primers used in this study. (DOCX)

S1 Fig Cell morphology in response to Hsp90 depletion. Deletion of *TEC1*, *MIG1*, or *UPC2* does not affect cellular morphology. Deletion of *TUP1* induces filamentation independent of Hsp90 levels. Strains were grown for six hours in non-filament inducing conditions (YPDM, 30°C). Images were captured using the Differential Interference Contrast setting on the Zeiss Axio Imager.MI microscope together with Axiovision software (Carl Zeiss, Inc.).
(TIF)

S2 Fig Cell length distribution in response to Hsp90 depletion in *BCR1* mutant and wild-type strains. Dot plot representing the lengths of cells and filaments in strains with genetically reduced Hsp90 and deletions of *BCR1*. Twenty randomly selected cells and filaments were scored for each strain and variance in length assessed. The *MAL2p-HSP90/hsp90 bcr1/bcr1* strain displayed significantly different longer cells (p<0.001).
(TIF)
