# Supplementary material for: Functional Divergence of Hsp90 Genetic Interactions in Biofilm and Planktonic Cellular States
Source: PLoS One. 2015 Sep 14;10(9):e0137947. doi: 10.1371/journal.pone.0137947 (PMC4569550; doi:10.1371/journal.pone.0137947)
Supplement: S1 Table — (DOCX) [file pone.0137947.s001.docx]

| **Strain ID** | **Name** | **Genotype** | **Reference** |
| --- | --- | --- | --- |
| CaLC239 | SN95 | *arg4∆/arg4∆* *his1∆/his1∆ URA3/ura3::imm434 IRO1/iro1::imm434* | [1] |
| CaLC432 | MAL2p-HSP90 | *arg4∆/arg4∆ his1∆/his1∆ URA3/ura3::imm434 IRO1/iro1::imm434, HIS1/his1::TAR-FRT, CdHIS1::hsp90/NAT-MAL2-HSP90* | [2] |
| CaLC2421 | SN95 bcr1∆ | As SN95 + *BCR1/bcr1∆* | This study |
| CaLC2676 | SN95 bcr1∆/∆ | As SN95 + *bcr1∆/∆* | This study |
| CaLC2423 | SN95 mig1∆ | As SN95 + *MIG1/mig1∆* | This study |
| CaLC2450 | SN95 mig1∆/∆ | As SN95 + *mig1∆/∆* | This study |
| CaLC2427 | SN95 tec1∆ | As SN95 + *TEC1/tec1∆* | This study |
| CaLC2560 | SN95 tec1∆/∆ | As SN95 + *tec1∆/∆* | This study |
| CaLC2429 | SN95 tup1∆ | As SN95 + *TUP1/tup1∆* | This study |
| CaLC2689 | SN95 tup1∆/∆ | As SN95 + *tup1∆/∆* | This study |
| CaLC2431 | SN95 upc2∆ | As SN95 + *UPC2/upc2∆* | This study |
| CaLC2453 | SN95 upc2∆/∆ | As SN95 + *upc2∆/∆* | This study |
| CaLC2613 | MAL2p-HSP90 bcr1∆ | As MAL2p-HSP90 + *BCR1/bcr1∆* | This study |
| CaLC2669 | MAL2p-HSP90 bcr1∆/∆ | As MAL2p-HSP90 + *bcr1∆/∆* | This study |
| CaLC2609 | MAL2p-HSP90 mig1∆ | As MAL2p-HSP90 + *MIG1/mig1∆* | This study |
| CaLC2717 | MAL2p-HSP90 mig1∆/∆ | As MAL2p-HSP90 + *mig1∆/∆* | This study |
| CaLC2608 | MAL2p-HSP90 tec1∆ | As MAL2p-HSP90 + *TEC1/tec1∆* | This study |
| CaLC2663 | MAL2p-HSP90 tec1∆/∆ | As MAL2p-HSP90 + *tec1∆/∆* | This study |
| CaLC2604 | MAL2p-HSP90 tup1∆ | As MAL2p-HSP90 + *TUP1/tup1∆* | This study |
| CaLC2719 | MAL2p-HSP90 tup1∆/∆ | As MAL2p-HSP90 + *tup1∆/∆* | This study |
| CaLC2600 | MAL2p-HSP90 upc2∆ | As MAL2p-HSP90 + *UPC2/upc2∆* | This study |
| CaLC2657 | MAL2p-HSP90 upc2∆/∆ | As MAL2p-HSP90 + *upc2∆/∆* | This study |
| CaLC2625 | SN95 TUP1-TAP | As SN95 + *TUP1/TUP1-TAP* | This study |
| CaLC2686 | MAL2p-HSP90 TUP1-TAP | As MAL2p-HSP90 + *TUP1/TUP1-TAP* | This study |

**References:**

1. Noble SM, Johnson AD. Strains and strategies for large-scale gene deletion studies of the diploid human fungal pathogen *Candida albicans*. Eukaryot Cell. 2005;4: 298–309.

2. Shapiro RS, Uppuluri P, Zaas AK, Collins C, Senn H, Perfect JR, et al. Hsp90 orchestrates temperature-dependent *Candida albicans* morphogenesis via Ras1-PKA signaling. Curr Biol. 2009;19: 621–629.
