# Supplementary material for: Functional Divergence of Hsp90 Genetic Interactions in Biofilm and Planktonic Cellular States
Source: PLoS One. 2015 Sep 14;10(9):e0137947. doi: 10.1371/journal.pone.0137947 (PMC4569550; doi:10.1371/journal.pone.0137947)
Supplement: S2 Table — (DOCX) [file pone.0137947.s002.docx]

| **Name** | **Primer ID** | **Sequence 3’ to 5’** |
| --- | --- | --- |
| **Transcription factor gene deletions by replacement with the NAT cassette** | | |
| BCR1_-70F | oLC2482 | ATAATAACTTAAATTTTCAAAAACAACAACTACAACAACTATAATTAATAATACTATCTCGATATATATC**GGAAACAGCTATGACCATG**^a^ |
| BCR1_+70R | oLC2483 | GACATAAGCAAATAAATAAATAAATAAATAAAAATACATTTTTCACATACTTTCTTCTTATTATTATTGT**GTAAAACGACGGCCAG** |
| BCR1_-140F | oLC2484 | CCCCTGAAAATTTGTCTCCTTCAATCAAACGATCAAATTATTCTATACGGTTGTTTGAAGTTGTTTATAT**GGAAACAGCTATGACCATG** |
| BCR1_+140R | oLC2485 | TACTCAGTTTATATAACAAACGAGTAAAGTAAGAACACTATAAAAAAGAAACAACATCAAAAACATCACA**GTAAAACGACGGCCAG** |
| BCR1_-605F | oLC2504 | TTTTCTCCAGTGACAACTTTTC |
| BCR1_+2764R | oLC2540 | GAGGAGCTATCGTATGACAC |
| MIG1_-70F | oLC2486 | CTTCTGTTTATTTCATTTTCATTTTCACTATACAAAACTAGACTTATTAATCAAGTTAATCAACGTCGCA**GGAAACAGCTATGACCATG** |
| MIG1_+70R | oLC2487 | TACCCAAAAAATCTATCTAAACCGTATTGTAAATCTATCTATAAATTAAAACACCAGTAATTATAAATCT**GTAAAACGACGGCCAG** |
| MIG1_-140F | oLC2488 | TTCCCCATCTTGAATTACCACGGAGAAAGAGATTGGAATAGAAAAAGAAAGACATTTCACCTTCAACTAA**GGAAACAGCTATGACCATG** |
| MIG1_+140R | oLC2489 | ATCTATATTACTAGTCTACTTGAATACCATTCATATATCATGCTTCTATTAAAAGAAAAAAGAACCTATA**GTAAAACGACGGCCAG** |
| MIG1_-308F | oLC2506 | CACCCAACGCATCCACACACAC |
| MIG1_+1890R | oLC2507 | CTAATTGACTTTCTGTACTTAC |
| TEC1_-70F | oLC2494 | TGTTTTTTTTTTTTCCCTTTTATAAATTATCCGTTAAGTTTACCGCTTTTTTTTTTGACTATTTTAAATT**GGAAACAGCTATGACCATG** |
| TEC1_+70R | oLC2495 | AGCCGCTAAACTAATGTATCCAACAGTGAGTAAGTGATAATATTTTCTTCTCTTTTCTCATTTTGTTCAC**GTAAAACGACGGCCAG** |
| TEC1_-140F | oLC2496 | TTATCGAACTTATCACGTGGTTGCTGTCAATTCCTATTTTTGTTTGCAATCTCACATTTGTTTGTTTGTT**GGAAACAGCTATGACCATG** |
| TEC1_-140R | oLC2497 | AAAAACACCAAATCAATTATGAACAAGATGAACCAAAGCTTCATCATCATTTACAATCGAACCAATAAAA**GTAAAACGACGGCCAG** |
| TEC1_-183F | oLC2510 | TTTGAATAGACTGTGGTATC |
| TEC1_+2570R | oLC2511 | TATGAAACAAGCAATTACAAC |
| TUP1_-70F | oLC2498 | AGCGCACCCCCTGTTCAAAAAAACCAACGAAAAAACAACACAACTTCTTCCATCCCCACCAGCAATGTCC**GGAAACAGCTATGACCATG** |
| TUP_+70R | oLC2499 | TGGGTTGTTACGAAAAAAATTTTTTTTTTTGGTGGTGTTTTTTTTTCTCACATATATATATACTACACAC**GTAAAACGACGGCCAG** |
| TUP1_-265F | oLC2512 | CTAAACATTGTCACTACATTC |
| TUP1_+1758R | oLC2513 | TTGGGATAAGGTGACGGAGATG |
| UPC2_-70F | oLC2500 | GAATCACAGTGAAGTTCTTTGAATCAAGTTCATTGAAGATATAGTTTTCAACCACTATTACTACCTTTCT**GGAAACAGCTATGACCATG** |
| UPC2_+70R | oLC2502 | GAAACCCGGCTAGTGTTGTAATAAACCCTACACAGTCGTAAATTCCTATCATCTACGCGGTATTGACCTG**GTAAAACGACGGCCAG** |
| UPC2_-140F | oLC2501 | TTAAACACTTTTTTCTTTCCCTTTTTCTTTAATTTATTACTACATCAAGTTTATTTATACCATAACTGCA**GGAAACAGCTATGACCATG** |
| UPC2_+140R | oLC2503 | TTTGTTATTAATCTCAAAACTAGCTGTGATTTTGATAAAGTACAATATCCCTTCTTTTGCAAATATCGGT**GTAAAACGACGGCCAG** |
| UPC2_-247F | oLC2514 | TTTATCAGATTGCCATAGCCAC |
| UPC2_+2448R | oLC2515 | ATTACACTACTTTCGTTTCC |
| pJK863down-F | oLC274 | CTGTCAAGGAGGGTATTCTGG |
| pJK863up-R | oLC275 | AAAGTCAAAGTTCCAAGGGG |
| **Verification of presence of transcription factor wild-type allele** | | |
| BCR1_+73F | oLC2516 | GACAACCAATGATGTATCCACC |
| BCR1_+573R | oLC2517 | GACAAGTAAAAGGTCGTTCACC |
| MIG1_+105F | oLC2518 | TGTGATAAGGCTTTCCATAG |
| MIG1_+589R | oLC2519 | GCTGTTGTTGATGTTGTGGAC |
| TEC1_+69F | oLC2522 | CTTCCATTAATTGTTGATGTTG |
| TEC1_+517R | oLC2523 | TGGACACGTGAATAAGCACCAG |
| TUP1_+70F | oLC2524 | ACGCCTCAAACGAAGCAAGCAG |
| TUP1_+616R | oLC2525 | AACTTGCCGTCTCTGGAAAATC |
| UPC2_+72F | oLC2526 | AAGACAAATAACTCTGAGCCTC |
| UPC2_+580R | oLC2527 | GAAGCTAAATTGGATAGACTGG |
| **TUP1 TAP tagging using pFA-TAP-ARG (pLC573)** | | |
| TUP1_TAP_F | oLC2568 | GGGAACCGAAGGTATCTTCGCTACAGGTAGTGGCGATTGTAAAGCCAGAATTTGGAAATGGACCAAAAAA**GGTCGACGGATCCCCGGGTT** |
| TUP1_TAP_R | oLC2569 | TGGGTTGTTACGAAAAAAATTTTTTTTTTTGGTGGTGTTTTTTTTTCTCACATATATATATACTACACAC**TCGATGAATTCGAGCTCGTT** |
| TUP_+1218F | oLC2570 | TTAGACAGAACCGTCAAGTTG |
| TAP-R | oLC1593 | TAAACTTTGGATGAAGGCG |
| ARG4-F | oLC1594 | ATGTTGGCTACTGATTTAGCTG |
| **Transcription factor RT-PCR** | | |
| BCR1_+244R | oLC2541 | GTGGTGGAAGTGGTGGCTGTC (with oLCxyz) |
| MIG1_+305R | oLC2542 | AGTTGTAGCTGTAGTAGCATTG |
| TEC1_+235R | oLC2544 | AAGTCTTTCTAATGGGAGTTC |
| TUP1_+249R | oLC2545 | ATTTGCCTGTCTCTAGTGTC |
| UPC2_+294R | oLC2546 | TGATGGGGGTTCATTATTGGC |
| PHO85_+120F | oLC2705 | GGGTGAAGGTACTTATGCCAC |
| PHO85_+285R | oLC2706 | CTCAGTGTGGATCACATCATAC |
| PCL1_+96F | oLC2707 | GTTATCCCTTGTGAAGACACC |
| PCL1_+276R | oLC2708 | AGTACAAGGTAATCCATGAGC |
| HMS1_+266F | oLC2709 | TGGTTTAGCTCCTCCTCCATT |
| HMS1_+433R | oLC2710 | AAGCACCAATTTCAGCGATAA |
| GPD1+570-F | oLC752 | AGTATGTGGAGCTTTACTGGGA |
| GPD1+766-R | oLC753 | CAGAAACACCAGCAACATCTTC |
| HSP90_+1051F | oLC756 | GCTGAAGAGTTGATTCCAGAAT |
| HSP90_+1236R | oLC757 | GGAGAAAGCAGTGTAGAATTGG |

________________________________________________________________________

^a^ sequence in bold is homologous to NAT cassette in pLC49
