# Supplementary figures and images for: Functional Divergence of Hsp90 Genetic Interactions in Biofilm and Planktonic Cellular States
Source: PLoS One. 2015 Sep 14;10(9):e0137947. doi: 10.1371/journal.pone.0137947 (PMC4569550; doi:10.1371/journal.pone.0137947)

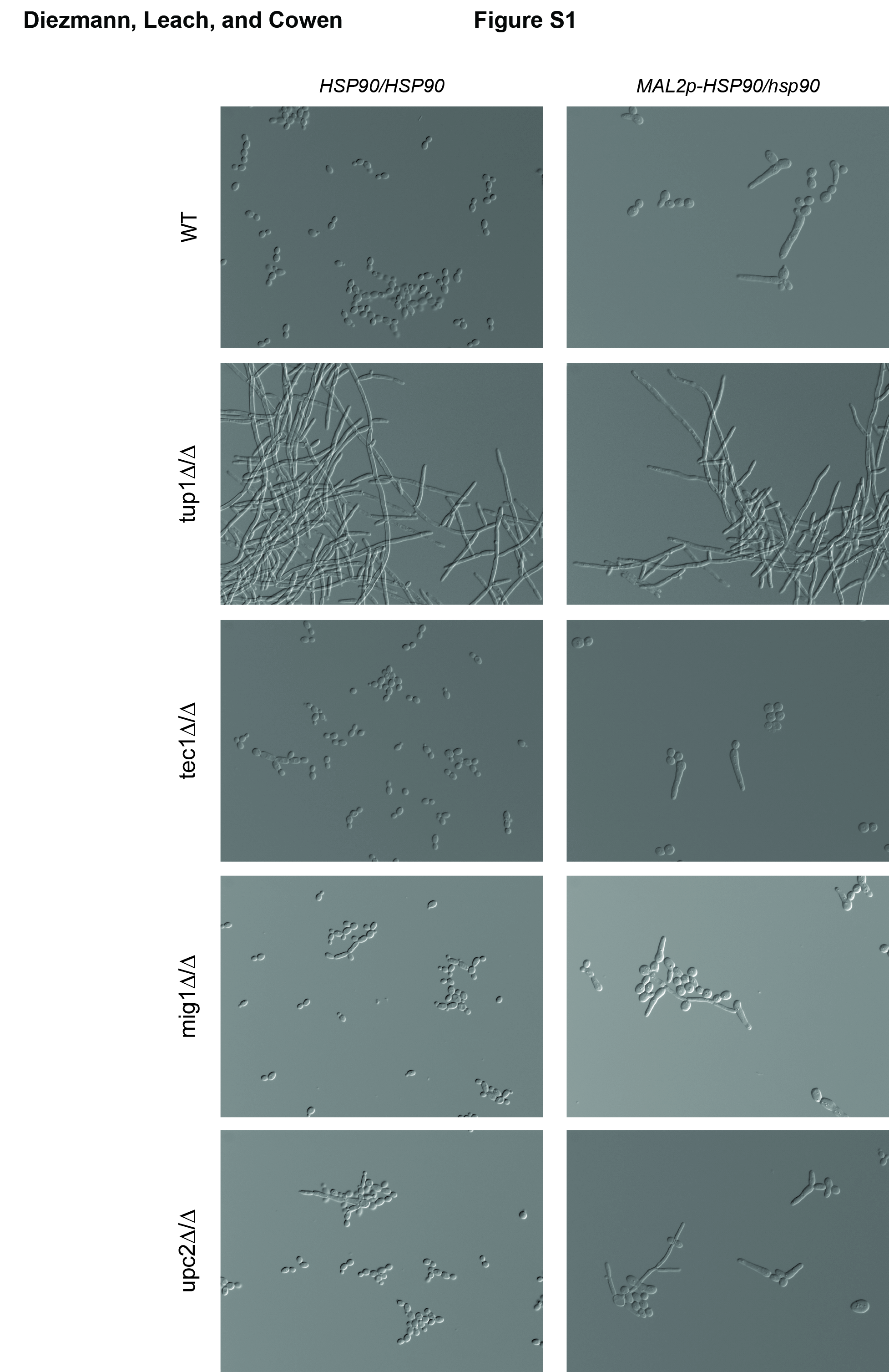

Supplement: S1 Fig — Deletion of TEC1, MIG1, or UPC2 does not affect cellular morphology. Deletion of TUP1 induces filamentation independent of Hsp90 levels. Strains were grown for six hours in non-filament inducing conditions (YPDM, 30°C). Images were captured using the Differential Interference Contrast setting on the Zeiss Axio Imager.MI microscope together with Axiovision software (Carl Zeiss, Inc.). (TIF) [file pone.0137947.s003.tif]

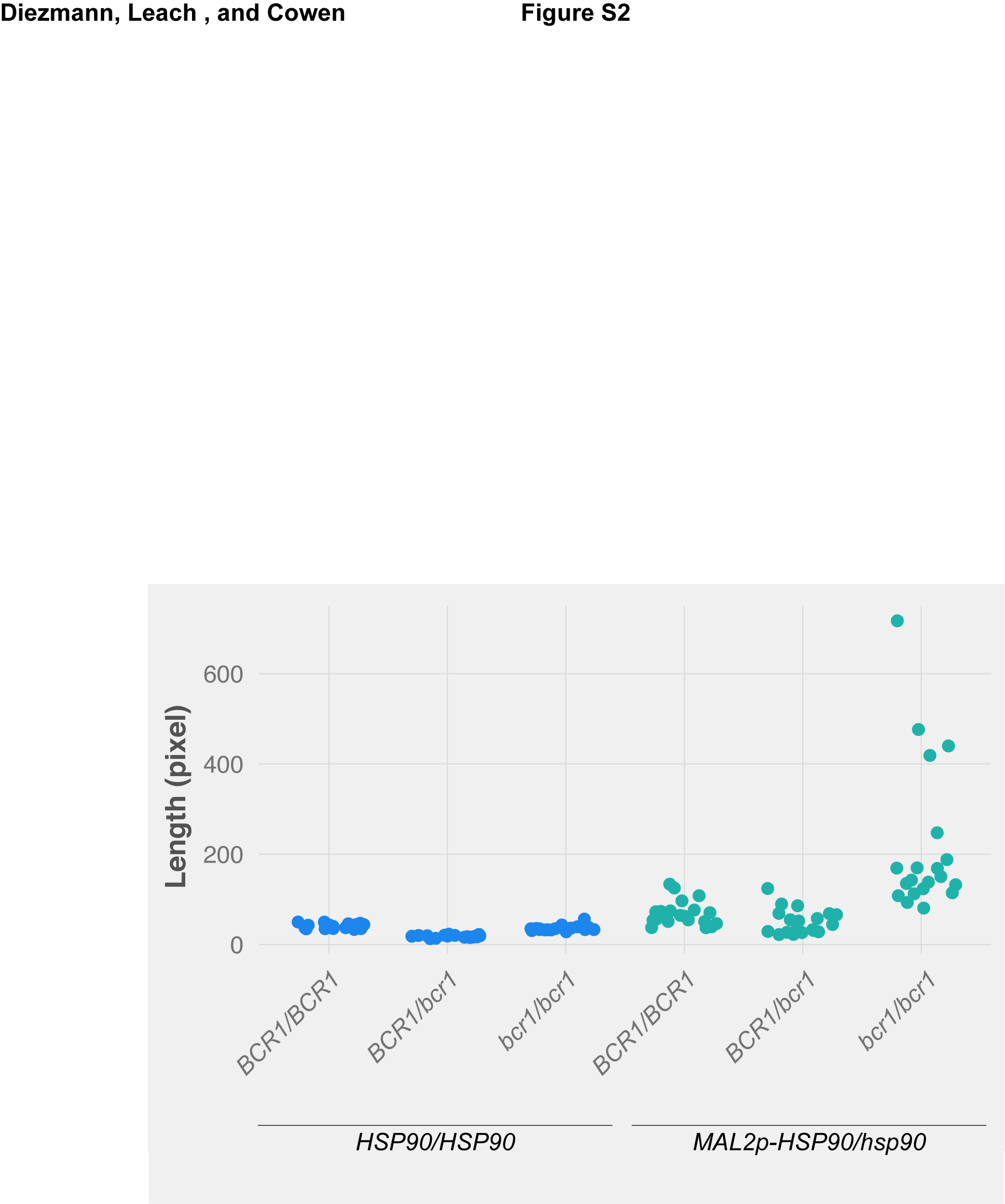

Supplement: S2 Fig — Dot plot representing the lengths of cells and filaments in strains with genetically reduced Hsp90 and deletions of BCR1. Twenty randomly selected cells and filaments were scored for each strain and variance in length assessed. The MAL2p-HSP90/hsp90 bcr1/bcr1 strain displayed significantly different longer cells (p<0.001). (TIF) [file pone.0137947.s004.tif]
